# Three-dimensional Mapping: More Than “Burn as You Learn”

**DOI:** 10.19102/icrm.2018.090505

**Published:** 2018-05-15

**Authors:** Rahul N. Doshi

**Affiliations:** ^1^Division of Cardiology, Keck USC School of Medicine, Los Angeles, CA, USA

**Keywords:** Atrial fibrillation, atrial flutter, 3D mapping, three-dimensional mapping

It has been 50 years since the beginning of invasive electrophysiology. Prior to the advent of catheters capable of recording electrical activity within the heart, electrophysiologic mechanisms were elucidated instead by careful observation of the electrocardiogram. One only needs to examine the revolutionary work of Katz and Pick^1^ to be truly humbled. However, the introduction of intracardiac recordings confirmed and further increased our understanding of arrhythmia mechanisms. Durrer et al.^2^ and Coumel et al.^3^ first demonstrated the critical physiology of Wolff-Parkinson-White syndrome in 1967. Scherlag et al.^4^ reported the first recording of the His bundle in 1969. Rosen et al.^5^ discussed dual atrioventricular nodal physiology for the first time in 1974. Beginning with these breakthroughs, intracardiac recordings and pacing maneuvers have become critical in our ability to differentiate types of supraventricular tachycardias; indeed, the sentinel paper by Knight et al.^6^ remains required reading for all electrophysiology fellows. From early observations to current clinical practice, intracardiac recordings are the cornerstone of understanding the mechanisms of supraventricular tachycardias.

## Enter the era of three-dimensional mapping

Since the introduction of modern mapping technologies into clinical practice,^7^ their use has rapidly expanded the type of arrhythmias that can be identified by fully integrating electrogram information with cardiac anatomy. However, much of current practice has shifted to a wholly anatomic approach. This is, of course, sometimes because of the arrhythmia itself—most notably, in the case of atrial fibrillation. From the early 2000s, three-dimensional mapping has proven to be a critical component for the ablation of atrial fibrillation^8^ without necessarily using the electrical information of the arrhythmia itself. Thus, the “burn as you learn” era began.

Catheter ablation for atrial fibrillation has created a need for mapping and ablation for postablation atypical flutters. These arrhythmias are often challenging to manage clinically. In this issue of *The Journal of Innovations in Cardiac Rhythm Management*, Sundaram et al.^9^ present an elegant case of three-dimensional mapping of an atypical atrial flutter with an alternating activation sequence in the coronary sinus, in which it was difficult to delineate the mechanism with traditional entrainment maneuvers. Via careful and detailed mapping, they proposed the following as an explanation for the alternating changes in activation sequence and cycle length: that they were due to the atrial myocardium in the potential critical isthmus reaching relative refractoriness shifting activation around the mitral annulus.

The use of three-dimensional mapping for atypical flutter is well-established^10^ and involves careful observations of the macroreentrant circuit and potential zone of slow conduction. Sundaram et al.^9^ hypothesize that, given the alternating circuit with an increase in cycle length corresponding to an increase in the path of propagation, the area of activation shift must reach refractoriness and thus must be an area of slow conduction. Ablation in this area resulted in arrhythmia termination and subsequent noninducibility. The authors have previously reported their technique for the mapping of atypical flutters.^11^

While it is easy to rely on three-dimensional mapping as simply a tool for more accurate navigation, the case presented here demonstrates the potential utility of such technologies to improve our understanding of complex arrhythmias. Advancements such as rotor mapping, high-density mapping, and automated vector notation can help us to better understand complex atrial arrhythmias, much like how intracardiac recordings increased our knowledge beyond the electrocardiogram. These developments will enable future clinical electrophysiologists to treat patients more successfully and efficiently. “Learning” and “burning”—the best of both worlds.
